# Isolation and Characterization of *Cylindrospermopsis raciborskii* Strains from Finished Drinking Water

**DOI:** 10.3390/toxins12010040

**Published:** 2020-01-08

**Authors:** Carina Menezes, Elisabete Valério, Maria João Botelho, Elsa Dias

**Affiliations:** 1 Department of Environmental Health, National Institute of Health Dr. Ricardo Jorge (INSA), Av. Padre Cruz, 1649-016 Lisbon, Portugal; carina.menezes@insa.min-saude.pt (C.M.); elisabete.valerio@insa.min-saude.pt (E.V.); 2 Department of Sea and Marine Resources, Portuguese Institute for the Sea and Atmosphere (IPMA), Rua Alfredo Magalhães Ramalho, 6, 1495-006 Lisbon, Portugal; mjbotelho@ipma.pt; 3 Centre for the Studies of Animal Science (CECA), Institute of Agrarian and Agri-Food Sciences and Technologies (ICETA), Oporto University, 4051-401 Oporto, Portugal

**Keywords:** *Cylindrospermopsis raciborskii*, finished water, saxitoxin, culture collection

## Abstract

In the summer of 2015, an intense cyanobacterial bloom producing geosmin/2-methylisoborneol (MIB) occurred in the Roxo freshwater reservoir in Alentejo, Portugal. The drinking water supplied from the Roxo water treatment plant (WTP) exhibited an unpleasant odor/taste and a significant cyanobacteria density was detected in the finished water at the exit of the WTP. Cyanobacteria were not evaluated downstream of the WTP, namely, at the city reservoir. The aim of this work was to isolate and characterize viable cyanobacteria present in finished water (exit of the WTP and city reservoir) that withstand conventional water treatment. Treated water samples collected at both sites were inoculated in Z8 culture medium to provide the conditions for putative cyanobacterial growth. After 30 days, filamentous cyanobacteria were observed in cultures inoculated with samples from the exit point of the WTP. Viable trichomes were isolated and identified as *Cylindrospermopsis raciborskii* by morphometric and molecular analysis. None of the isolates were cylindrospermopsin/microcystin producers, as confirmed by ELISA and amplification of corresponding genes (*PS*/*PKS* and *mcyA-cd*/*mcyAB*/*mcyB*). ELISA results were positive for saxitoxin, but saxitoxin and derivatives were not detected by liquid chromatography with fluorescence detection (LC-FLD), nor were their related genes (*sxtA*/*sxtA4*/*sxtB*/*sxtM*/*sxtPer*/*sxtI*). To our knowledge, this is the first report on the establishment of cultures of *C. raciborskii* that resisted water treatment processes.

## 1. Introduction

The Roxo reservoir is a freshwater source located in Alentejo, south Portugal that is used to supply drinking water for the municipalities of Beja (35,854 inhabitants) and Aljustrel (4600 inhabitants). It is also used for irrigation, since it is located in a rural area with important agricultural activity. This region, characterized by a Mediterranean climate, is particularly vulnerable to climate change, namely, severe and prolonged droughts [1,2]. These conditions have a negative impact on water availability and may favor eutrophication and cyanobacteria blooming [3]. Indeed, the Roxo reservoir is classified as hypertrophic [4,5] and has a long history of cyanobacterial blooms [6,7].

In the summer of 2015, an intense bloom composed of a great diversity of filamentous cyanobacteria (*Aphanizomenon* spp., *Planktothrix* spp., *Cylindrospermopsis raciborskii*, and *Anabaena* spp.) occurred in the Roxo reservoir [6,8]. Cell density in raw water ranged between 52,000 and 108,000 cells/mL from July to September [6]. Several cyanotoxins (microcystin, saxitoxin (STX), and cylindrospermopsin) were detected in raw and treated water although at levels below the corresponding national [6,8] and international regulatory and/or guideline values [9]. This persistent bloom caused an intense odor in raw water due to the release of geosmin and 2-methylisoburneol (MIB) [6,8]. The unpleasant taste and odor was detected in consumers’ tap water as well [6,8]. Additionally, cyanobacterial cells (up to 1000 cells mL^−1^) were also detected in treated water samples collected at the exit point of the water treatment plant (WTP) [6,8]. The quality control plan of the Roxo reservoir did not include the phytoplankton monitoring of water in city reservoirs, so it was unknown if cyanobacterial cells were present in high densities downstream of the WTP.

Interestingly, *C. raciborskii* was one of the dominant species of the cyanobacterial community in the Roxo reservoir. This species settled at this reservoir in 2015 and became a resident species, at least until 2018 [6]. Indeed, once considered a tropical cyanobacterium, *C. raciborskii* is nowadays considered a cosmopolitan species, found not only in tropical regions but also in temperate areas [10]. Several particularities of *C. raciborskii* have enabled the success of this species and its ability to spread and colonize diverse water environments. Besides the features that it shares with other cyanobacteria, such as the development of akinetes and heterocytes, buoyancy regulation, and grazing resistance, *C. raciborskii* has a high affinity for phosphorus (P) and P storage, a tolerance to low light and salinity, allelopathic activity, and phenotypic plasticity [10,11]. The occurrence of *C. raciborskii* blooms in freshwater reservoirs, used for recreational activities and/or as a source of drinking water, constitutes a public health concern due to the ability of this species to produce cylindrospermopsin [12,13] and paralytic shellfish toxins [14,15]. Recently, microcystin production by *C. raciborskii* was also hypothesized [16].

Until 2016, conventional water treatment processes were used at the Roxo WTP, namely, coagulation, flocculation, sedimentation, and filtration. It has been reported that these approaches might not be efficient when a reservoir presents high cyanobacterial densities [17,18,19]. Although most of the studies on water treatment methods focus on the elimination of cyanotoxins, it is also important to consider the removal of the intact cyanobacterial cells, even of nontoxic species, considering that they can release other intracellular compounds that have an impact on water quality [17,18]. Indeed, besides cyanotoxins, taste and odor compounds can be released from intact cells that breakthrough the WTP. This poses water quality issues because, unlike several cyanotoxins, these compounds have been shown to be resistant to most disinfectants [20]. Therefore, the choice of water treatment methods should be performed on a case-by-case basis in order to adapt the various existing methodologies to the organisms and their compounds [19,21]. This is particularly true for the Roxo reservoir, considering that the inclusion in 2016 of advanced treatment methods at the WTP considerably enhanced the efficiency of removal of cyanobacteria and related compounds [6]. The incorporation of an ozonation stage and granulated activated carbon (GAC) filters increased the elimination/degradation of organic compounds and the removal/inactivation of cyanobacteria cells [21,22,23].

The present work aimed to (1) isolate viable cyanobacteria from treated water samples collected at the exit point of the Roxo WTP and the city (Beja) distribution reservoir and establish monoalgal cultures of those isolates, and (2) characterize those isolates regarding their morphometry, taxonomy, and toxin production.

## 2. Results

### 2.1. Phytoplankton Composition of Treated Water Samples

The phytoplankton species and the corresponding cell densities of the treated water samples are shown in Table 1. Besides very low densities of diatoms and chlorophytes (less than 2%), a high cyanobacterial density (2514 and 1613 cells mL^−1^) was detected both at the exit point of the WTP and at the city reservoir. According to the identification keys described in EN 15204:2006 [24], cyanobacteria were identified in both samples as *C. raciborskii* and *Pseudanabaena limnetica*. In the sample collected at the city reservoir, *Aphanizomenon gracile* was also detected.

### 2.2. Isolation of Viable Cyanobacteria from Treated Water Samples

Phytoplankton growth was observed 30 days after the inoculation of filtered water samples from both sampling points in Z8 medium. In both cases, the presence of chlorophytes and diatoms was observed. Additionally, *C. raciborskii* trichomes were present in cultures obtained from the sample collected at the exit point of the WTP. Three monoalgal cultures of this species were established. These isolates are hereinafter referred to as LMECYA 324, LMECYA 325, and LMECYA 326.

### 2.3. Morphological and Molecular Characterization of C. raciborskii Isolated from Treated Water Samples

**Figure 1:**
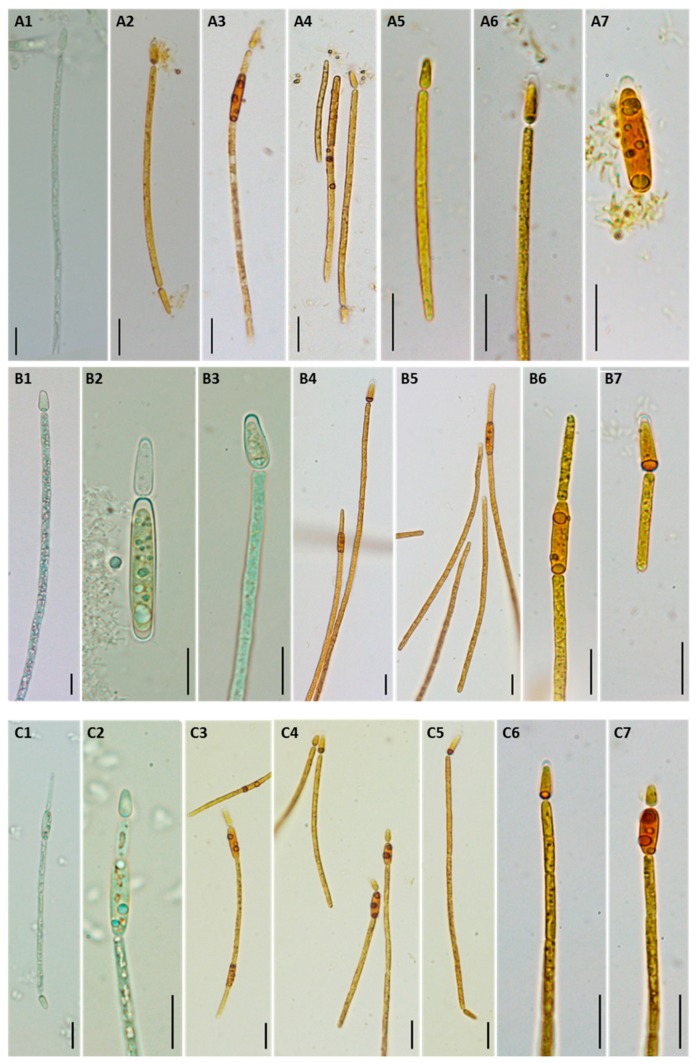
Morphological characteristics of *C. raciborskii* isolates LMECYA 324 (**A**), LMECYA 325 (**B**), and LMECYA 326 (**C**). Microphotographs from live (A1, B1–3 and C1–2) and Lugol’s-fixed cultures (A2–7, B4–7 and C3–7). Magnification 400× (A1–4, B1, B4–5, and C1, C3–5) and 1000× (A5–7, B2–3, B6–7 and C2, C6–7). Scale bar = 10 µm.

The three *C. raciborskii* isolates exhibited mainly straight trichomes (Figure 1(A1,A4–A6,B3,B6,B7,C1,C5–C7)), and only a small fraction of these were slightly curved (Figure 1(A3,B5,C3)). Heterocytes were mostly found at one end of the filaments, although some isopolar trichomes were also observed (Figure 1(A2,C5)). Akinetes developed in the following cell or close to the heterocyte (Figure 1(A3,B5,B6,C2–C4,C7)).

**Figure 2:**
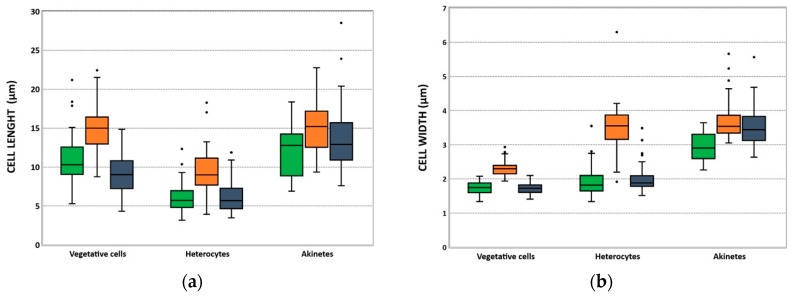
Distribution of the length (µm) (**a**) and width (µm) (**b**) of vegetative cells, heterocytes, and akinetes from *C. raciborskii* LMECYA 324 (green), LMECYA 325 (orange), and LMECYA 326 (violet). The box boundaries indicate the 25th and 75th percentiles. The line within the box marks the median and the black dots indicate outliers.

Length and width of vegetative cells, heterocytes, and akinetes (Table 2 and Figure 2) displayed values within the ranges previously described for *C. raciborskii* [25,26,27,28]. Of the three isolates, LMECYA 325 presented cells with higher dimensions compared with LMECYA 324 and LMECYA 325 (Table 2 and Figure 2), suggesting that it is apparently distinct from the other two isolates.

**Figure 3:**
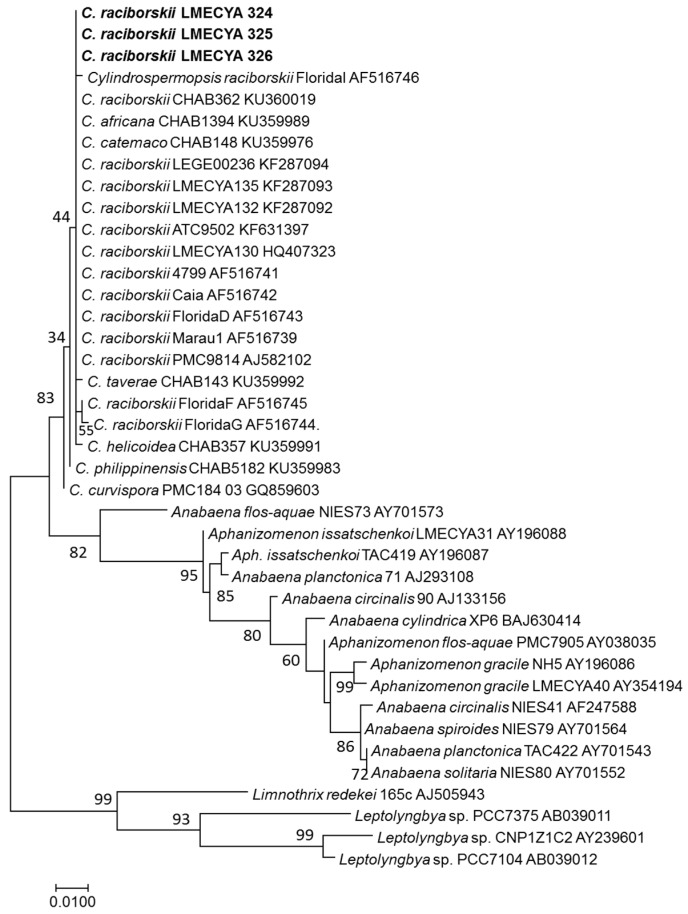
Phylogenetic tree of *C. raciborskii* LMECYA 324–326 and related taxa obtained by the maximum likelihood method of the 16S rDNA. An alignment of 540 positions in the final dataset was used. Percentage bootstrap values of 1000 replicates are given at each node. GenBank accession numbers are indicated after species designation (names in bold-face correspond to the sequence determined in this study).

The 16S rDNA gene sequences obtained from the cultured strains showed a 99.64% similarity with several *C. raciborskii* strains, and the phylogenetic analysis (Figure 3) showed that LMECYA 324, LMECYA 325, and LMECYA 326 clustered together with all the other *C. raciborskii* strains, thus confirming the identity of these isolates.

### 2.4. Evaluation of C. raciborskii Toxicity

The genes involved in the biosynthesis of cylindrospermopsin, saxitoxin, and microcystin were not detected in LMECYA 324, LMECYA 325, and LMECYA 326 cultures (Table 3). The results from the ELISAs confirmed the inability of the isolates to produce cylindrospermopsin and microcystins (Table 3). For saxitoxin, the ELISA performed in the LMECYA 324 and LMECYA 325 isolates gave a positive result of 0.02 µg L^−1^. Because this is the limit of quantification of the method, the assay was repeated in concentrated cultures of the three isolates with results of 0.20 µg L^−1^ for LMECYA 324, 0.08 µg L^−1^ for LMECYA 325, and 0.07 µg L^−1^ for LMECYA 326. By applying the corresponding dilution factor, the saxitoxin concentrations in the *C. raciborskii* cultures were 0.0178 µg L^−1^ for LMECYA 324, 0.0070 µg L^−1^ for LMECYA 325, and 0.0052 µg L^−1^ for LMECYA 326. To identify the putative saxitoxin and potential analogues, liquid chromatography with fluorescence detection (LC-FLD) analysis was performed, but the results for the extracts of the three isolates were below the quantification limit for all the saxitoxin derivatives analyzed (STX, C1 + 2, dcSTX, GTX5, dcGTX2 + 3, dcNEO, GTX1 + 4, GTX2 + 3, and NEO (Table 3)).

## 3. Discussion

In this paper, we described the isolation and culturing of *C. raciborskii* isolates obtained from finished water samples. The treated water samples were collected at the exit point of the WTP of the Roxo reservoir (Alentejo, Portugal), located in a region particularly susceptible to very warm temperatures and severe droughts, such as those registered since 2015 [1,2]. It is known that *C. raciborskii* is spreading to temperate regions [10]; thus, it is not surprising that the occurrence of this species is increasing, particularly in south Europe, where the impact of climate change and global warming is already perceptible [29,30]. In fact, occurrences of *C. raciborskii* were already reported in surface freshwater reservoirs from south Portugal [31,32].

Indeed, the increase of bloom frequency and intensity registered in the last few years in the Roxo reservoir [6,7] has been compromising the water quality from this important source of freshwater in the region. Although residual concentrations of cyanotoxins were detected in treated water (below national and international regulatory/guideline levels [6,9]), the number of cyanobacterial cells was often very high [8], which constitutes a potential health risk. Besides, if blooming species are able to produce taste and odor compounds, the organoleptic quality of the water can also be compromised, as in the episode of 2015.

The situation of the Roxo reservoir led to the implementation in 2016 of measures to improve water treatment processes, extending the existing conventional methods [33]. The installation of an ozonation pretreatment step would allow a more efficient oxidation of organic matter. The efficiency of oxidation methods for water treatment depend on the sensitivity of cyanobacterial species to the specific oxidant. Although *C. raciborskii* has been described as very susceptible to chlorine oxidation [34], it is completely inactivated by ozonation, with the use of low concentrations of ozone (1 mg min^−1^ L^−1^) [35]. Furthermore, the installation of a filtration step with granular activated carbon would allow for effective adsorbance of odors and undesirable flavors, such as those registered in 2015 in the drinking water supplied to a population of 40,000 inhabitants. The implementation of these advanced treatment methods led to a marked decrease of cyanobacterial cells in the finished water, as shown in a monitoring study carried out during 2017/2018 [6]. On the other hand, attempts to reisolate living cells from samples of water treated with these new methods were unsuccessful.

Interestingly, in our study, *C. raciborskii* was predominant in treated water samples from the WTP of the Roxo reservoir, despite the presence of a great diversity of cyanobacteria in raw water (as referred to in the introduction), and only this species was able to survive to conventional water treatment and grow after inoculation in laboratory-controlled conditions. It was previously reported that the efficiency of cyanobacteria removal is species dependent and filamentous cyanobacteria, especially *Aphanizomenon* spp., are the most difficult to eliminate in conventional water treatments [18,36]. Eventually, we can extrapolate this observation to *Cylindrospermopsis* spp. This emphasizes the importance of suitable treatment methods considering that *C. raciborskii* resisted the water treatment and could have released noxious compounds downstream of the WTP along the water distribution system, potentially contaminating the water supplied to the populations. We should note, however, that viable *C. raciborskii* were only recovered from the samples collected at the exit point of the WTP and not from the city reservoir. The total absence of sunlight and the residual chlorine levels in the city reservoir might have completed impaired *C. raciborskii* cell viability.

In the present study, cylindrospermopsins and microcystins were not detected in *C. raciborskii* isolates. Additionally, LC-FLD methodologies did not confirm the positive results for saxitoxin obtained by ELISA. Although this may suggest that the ELISA results were false positives caused by matrix effects (due to the concentration of the cultures), we should consider the differences in the limits of quantification of these methods (LOQ_ELISA_ = 0.02 µg L^−1^; LOQ_LC-FLD_ = 1.9 µg L^−1^). Even though the genes associated with saxitoxin production (*sxtA*, *sxtA4*, *sxtB*, *sxtM*, *sxtPer*, and *sxtI*) were not detected, we should not disregard the possibility of a putative alternative pathway being responsible for STX production in these *C. raciborskii* isolates, although at a low level of expression.

So far, only a few *C. raciborskii* strains were isolated from Portuguese freshwater reservoirs and none were identified as toxin producers, as reported previously [31]. These authors hypothesized that the European cluster of *C. raciborskii* originated from the Asia/Australia isolates, which are described as cylindrospermopsin producers. Besides, only the South American *C. raciborskii* isolates have been identified as saxitoxin producers [10,31,37]. Thus, it might seem improbable that the Roxo isolates produce this toxin. Interestingly, segments from the *mcy* gene cluster were identified in a Tunisian *C. raciborskii* strain, and microcystin production was reported in a Greek isolate of this species [16,37]; however, the *mcy* genes tested were not present in the three isolates of this study.

*C. raciborskii* strains isolated from the Roxo WTP are presently maintained at the “Estela Sousa e Silva Algae Culture Collection” (ESSACC). In the scope of a large study concerning the characterization of this culture collection, we are sequencing the genomes (MiSeq Illumina^®^ platform) of cyanobacterial strains, including LMECYA 324, LMECYA 325, and LMECYA 326. The sequencing procedures and bioinformatics analysis are currently being performed and preliminary results corroborate the identification of the three isolates as *C. raciborskii*. With these tools, we hope to ascertain the potential toxicity of these isolates and clarify the doubts that have arisen with the present work. On the other hand, the genome analysis of the isolates might contribute to the knowledge of cyanobacterial features underlying their resistance to water treatment methods.

The presence of cyanobacteria in treated water, even though they are not toxin producers, might compromise the water quality because cyanobacterial species can produce taste and odor compounds [38] and chlorinated byproduct precursors [39]. The improvement and suitability of the procedures at the WTP, along with a robust monitoring program, is thus essential in order to reduce the impacts and potential risks posed by cyanobacterial blooms in drinking water sources.

## 4. Conclusions

The *C. raciborskii* cells remaining after the conventional water treatment processes at the WTP maintained the ability to grow and produce healthy cultures. Three strains (LMECYA 324, LMECYA 325, and LMECYA 326) of *C. raciborskii* were isolated from treated water samples and are maintained as monoalgal cultures at the ESSACC.

Despite the apparent inability of these strains to produce cylindrospermopsins, microcystins, and saxitoxins, the ability of *C. raciborskii* strains to resist water treatments should not be disregarded, considering that they can release noxious compounds downstream of the WTP.

To our knowledge, this is the first report of the establishment of cultures of *C. raciborskii* isolated from finished drinking water.

## 5. Materials and Methods

### 5.1. Water Sampling and Phytoplankton Analysis

Water samples (5 L) were collected at the exit point of the WTP and the distribution reservoir located in the city of Beja. The samples were transported under refrigeration to the laboratory. In both samples, aliquots of 100 mL were preserved with Lugol’s solution and phytoplankton species were analyzed and quantified according to the Utermöhl technique [24]. Briefly, 25 mL of each of the preserved and homogenized samples were used to fill a sedimentation chamber that was left to settle for 24 h, after which observation was performed by phase contrast optical microscopy (Olympus CK40). Taxonomic identification was based on microscopic observation of distinctive morphological features and identification keys according to annex D from [24].

### 5.2. Isolation of Cyanobacteria from Treated Water Samples

Aliquots of 100 mL of water samples from both sampling points were filtered under slight vacuum using membranes of 0.45 µm (MF-Millipore, Darmstat, Germany) in order to concentrate the phytoplankton biomass. The filters were then washed into a six-well microplate with Z8 culture medium [40] to inoculate the filtered cyanobacteria. These inocula were maintained under controlled culture conditions (20 ± 1 °C; 20 ± 4 μmol m^−2^ s^−1^; 14 h light/10 h dark cycle) at the ESSACC [41]. Cell growth was followed by optical microscopic examination (Olympus CK40). In the wells where cell growth was observed, cyanobacteria colonies were isolated with a stretched Pasteur pipette, transferred into new flasks containing Z8 medium, and maintained in the abovementioned temperature and light conditions. Three new isolated monoalgal, nonaxenic cultures were added to the ESSACC collection as LMECYA 324, LMECYA 325, and LMECYA 326.

### 5.3. Morphological Characterization of C. raciborskii Isolates

Morphometric characterization of *C. raciborskii* isolates from the abovementioned cultures, at the exponential growing phase, was performed by analyzing microphotographs of trichomes using an optical microscope (Olympus BX60) coupled with a CCD camera (Olympus DP11) at 400× and 1000× magnification. The following morphological features were determined: vegetative cell dimensions (length and width), trichrome shape, and presence and dimensions of heterocytes and akinetes. For the three cultures, measurements were performed with ImageJ V1.52A software [42] for at least 50 cells, with the exception of LMECYA 324, where it was only possible to measure 18 akinetes.

### 5.4. Molecular Identification of C. raciborskii Isolates by PCR Amplification and Sequencing of 16S rDNA Gene

The DNA extraction and purification from exponentially growing *C. raciborskii* isolates was done using the Invisorb^®^ Spin Plant Mini Kit (Invitek, Berlin, Germany), according to the manufacturer’s instructions. The concentration of DNA in samples was determined with a NanoDrop^®^ ND-1000 spectrophotometer (NanoDrop Technologies, Inc., Wilmington, DE, USA). The 260/280 and 260/230 nm ratios were also evaluated to assess the DNA purity. Samples were diluted to a final DNA concentration of 50–150 ng/µL.

The amplification and sequencing of the 16S rDNA gene was performed using primers CYAN106F (5′-CGGACGGGTGAGTAACGCGTGA-3′) [43] and CYAN738R (5′-GCTAGGACTACWGGGGTAT-3′) [44]. The PCRs were performed in a 50 µL mix containing 1× PCR buffer (Invitrogen, Waltham, Massachusetts, USA), 0.05 mM of each of the four dNTPs (Invitrogen), 0.25 mM of each primer, 50 ng of genomic DNA, 2 mM MgCl_2_ (Invitrogen), and 1 U Taq DNA polymerase (Invitrogen). Amplification was performed in a thermocycler (Biometra, Goettingen, Germany) using the following conditions: 10 min of initial denaturation at 95 °C, followed by 35 cycles at 94 °C for 45 s, 55 °C for 45 s, and 72 °C for 1 min. The amplification was completed by holding for 5 min at 72 °C to allow the complete extension of the PCR product.

The amplified products were separated in 1% (*w/v*) agarose gel electrophoresis in 0.5× TBE buffer, with 0.25 × GelRed (Biotium, Mannheim, Germany) incorporation, at 90 V for 45 min using the 1 kb plus DNA Ladder (Gibco-BRL, Waltham, MA, USA) as the molecular size marker and visualized by exposure to ultraviolet light.

PCR products were sequenced in both directions using BigDye terminators and the 16S rDNA primers mentioned above, according to standard protocols. The resulting chromatograms were read and edited using the BioEdit program.

The evolutionary history was inferred by using the maximum likelihood method based on the Tamura–Nei model [45]. The analysis involved 40 nucleotide sequences. All positions containing gaps and missing data were eliminated. There were a total of 540 positions in the final dataset. Evolutionary analyses were conducted in MEGA7 [46].

### 5.5. Quantification of Cyanotoxins in C. raciborskii Cultures

Specific ELISA kits were used to quantify total cylindrospermopsins and saxitoxins (CAT nos. 20-0149 and 20-0173, Beacon, Saco, ME, USA, respectively) and microcystins (CAT no. 520011, Abraxis, Warminstair, PA, USA) in exponentially growing cultures of *C. raciborskii* strains. Two independent aliquots of the cultures (5 mL) were frozen before the analysis. In the case of saxitoxin, specific kit buffer solutions were added to the samples before freezing the aliquots. Aliquots of *C. raciborskii* cultures were defrosted at room temperature (one freeze/thaw cycle) and sonicated (Sonics Vibracell V505 probe, Newtown, CT, USA) for 2 min (10 s sonication/2 s rest cycle) to release intracellular cyanotoxins. The assays were performed according to the manufacturer’s instructions. Absorbance was measured at 450 nm using a microplate ELISA photometer (Thermo, Labsystems Multiskan Ascent^®^, Helsinki, Finland) and acceptable sample variance was <15%. Toxin concentration was calculated from the average of the ELISA results of the two replicate samples and are given in micrograms of cyanotoxin (equivalents) per liter of water. These methods provide a quantification limit of 0.1, 0.02, and 0.15 µg L^−1^ for cylindrospermopsins, saxitoxins, and microcystins, respectively.

As the value of saxitoxins coincided with the quantification limit of the method, the ELISA procedure was repeated in concentrated samples. The concentration procedure was performed on a speed-vac system (Micromodul Y10, Savant, NY, USA). The concentration factor was 11.1 for LMECYA 324, 11.9 for LMECYA 325 and 12.6 for LMECYA 326.

### 5.6. Saxitoxin Derivatives Analysis by LC-FLD

Samples from LMECYA 324, LMECYA 325, and LMECYA 326 cultures were collected in order to analyze STX and potential analogues by LC-FLD. Culture samples were passed through GF/C glass filters (porosity 1.2 µm, 150 mm Ø) under light vacuum pressure (100 mmHg), and the material retained in the filters was frozen in 0.1 M acetic acid at −80 °C until analysis. Intracellular content was extracted from the biomass retained on the filters by the freeze/thaw cycle, followed by probe sonication (Vibra Cell, Sonics & Materials Inc., Newtown, CT, USA) in an ice bath (30 s, 60% amplitude, and 30 W) (adapted from [47]). Examination under an inverted microscope revealed the full disruption of the cells in the samples. The extracts passed through a solid-phase extraction C18 cartridge (500 mg/3 mL, Supelclean, Supelco, St. Luis, MO, USA) and pH was adjusted to 6.5 with 0.2 M NaOH. The extracts were filtered (0.2 µm nylon syringe filter) and diluted to exactly 1.5 mL. The determination of saxitoxins was based on the AOAC precolumn oxidation method by LC-FLD [48,49]. Aliquots of culture extracts were used for oxidation of saxitoxins with peroxide and periodate oxidant prior to LC-FLD analyses. A similar procedure for both oxidations was followed, replacing the oxidant reagent with water in order to detect naturally fluorescent compounds. The quality control of the results was assured by the use of the certified reference materials C1&2-b, STX-f, dcSTX-b, GTX5-c (B1), dcGTX2&3-c, dcNEO-d, GTX1&4-d, GTX2&3-c, and NEO-d from the Institute for Marine Biosciences, National Research Council Canada. Instrumental detection limits (nmol L^−1^) for individual toxins in C18-cleaned extracts were 4 (GTX2 + 3, GTX5, STX, and dcSTX), 20 (dcGTX2 + 3 and C1 + 2), 30 (NEO), and 40 (GTX1 + 4 and dcNEO). The LC system consisted of an Agilent Model 1290 Infinity II quaternary pump, an in-line degasser Model 1290 Infinity autosampler, and a Model 1260 fluorescence detector and column oven. The OpenLAB CDS software performed data acquisition and peak integration. The saxitoxin oxidation products were separated using a reversed-phase column Supelcosil LC-18, 150 × 4.6 mm i.d., 5 µm particle size (Supelco, St. Luis, MO, USA), equipped with a guard column Supelguard Supelcosil C18, 20 × 4.0 mm i.d., 5 µm particle size (Supelco). The column was kept in an oven at 30 °C. Two mobile phases were used for separation of saxitoxins: solution A (0.1 mol L^−1^ ammonium formate, pH = 6) and solution B (0.1 mol L^−1^ ammonium formate in 5% acetonitrile, pH = 6). The mobile-phase gradient used in chromatography consisted of 0–10% B in the first 4 min, 10–90% B in the next 5 min, back to 10% B in the next 2 min, and 0% B in the last 2 min. The flow rate was 1 mL min^−1^ and the injection volumes were 40 and 80 µL for the oxidation products of peroxide and periodate reaction, respectively. The excitation and emission wavelengths for fluorometric detection were set at 340 and 395 nm, respectively.

### 5.7. PCR Amplification of Genes Involved in Microcystin, Cylindrospermopsin, and Saxitoxin Production

The genes involved in microcystin production (*mcy*) were screened using a multiplex PCR for the simultaneous amplification of *mcyA-cd*, *mcyAB*, and *mcyB* gene fragments, performed according to a previously reported method [50].

For the amplification of genes involved in cylindrospermopsin production (*PS* and *PKS*), a multiplex PCR was also used, as previously described [51].

In the case of saxitoxin production, genes *sxtA*, *sxtA4*, *sxtB*, *sxtM*, *sxtPer*, and *sxtI* were tested according to the references listed in Table 4.

## Figures and Tables

**Table 1:** Phytoplankton profile and cell densities of water samples collected at the exit point of the water treatment plant (WTP) and the city reservoir. Groups of phytoplankton are displayed in bold.

Phytoplankton Species	Cell Densities (cells mL^−1^)	
	Exit Point of WTP	City Reservoir
**Cyanobacteria**		
*Cylindrospermopsis raciborskii* *Pseudanabaena limnetica* *Aphanizomenon gracile*	1472 1042 −	651 383 579
**Diatoms**		
*Nitzschia acicularis*	30	13
**Chlorophytes**		
*Scenedesmus ellipticus*	26	-
Total	2570	1627

**Table 2:** Dimensions of vegetative cells, heterocytes, and akinetes of *C. raciborskii* isolates LMECYA 324, LMECYA 325, and LMECYA 326. N refers to the number of measured cells.

Isolates	Vegetative Cell Length Mean ± SD (µm) (N)	Vegetative Cell Width Mean ± SD (µm) (N)	Heterocyte Length Mean ± SD (µm) (N)	Heterocyte Width Mean ± SD (µm) (N)	Akinete Length Mean ± SD (µm) (N)	Akinete Width Mean ± SD (µm) (N)
LMECYA 324	10.83 ± 3.22 (52)	1.74 ± 0.16 (50)	5.91 ± 1.62 (63)	1.90 ± 0.37 (64)	12.18 ± 3.01 (18)	2.94 ± 0.41 (18)
LMECYA 325	14.75 ± 2.61 (71)	2.29 ± 0.19 (71)	9.35 ± 2.54 (70)	3.49 ± 0.61 (70)	15.16 ± 3.36 (73)	3.68 ± 0.50 (70)
LMECYA 326	9.05 ± 2.25 (71)	1.73 ± 0.16 (73)	6.03 ± 1.95 (50)	1.98 ± 0.37 (50)	13.71 ± 4.08 (50)	3.50 ± 0.54 (53)

**Table 3:** Quantification of cyanotoxins by ELISA (microcystins, cylindrospermopsins, and saxitoxins) and by liquid chromatography with fluorescence detection (LC-FLD) (saxitoxin and derivatives) in *C. raciborskii* LMECYA 324, LMECYA 325, and LMECYA 326 cultures. Detection of cyanotoxin-related genes in these strains by PCR amplification.

Toxin Genes/Cyanotoxin	LMECYA 324	LMECYA 325	LMECYA 326
*mcyA-cd*/*mcyAB*/*mcyB* genes Microcystins (ELISA)	Negative <0.15 µg L^−1^	Negative <0.15 µg L^−1^	Negative <0.15 µg L^−1^
*PS*/*PKS* genes Cylindrospermopsin (ELISA)	Negative <0.1 µg L^−1^	Negative <0.1 µg L^−1^	Negative <0.1 µg L^−1^
*sxtA*/*sxtA4*/*sxtB*/*sxtM*/*sxtPer*/*sxtI* genes Saxitoxin	Negative 0.0178 µg L^−1^ (ELISA) <1.9 µg L^−1^ (LC-FLD)	Negative 0.0070 µg L^−1^ (ELISA) < 1.9 µg L^−1^ (LC-FLD)	Negative 0.0052 µg L^−1^ (ELISA) <1.9 µg L^−1^ (LC-FLD)

**Table 4:** PCR primer sequences for the amplification of genes involved in saxitoxin production and amplicon sizes.

Gene	Primers	Sequence (5′-3′)	Size (bp)	References
*sxtA*	jrtPKSF	GGAGTGGATTTCAACACCAGAA	147	Referred to in [52]
	jrtPKSR	GTTTCCCAGACTCGTTTCAGG		
*sxtA4*	SXTA4-F	GGACTCGGCTTGTTGCTTC	200	[53]
	SXTA4-R	CCAGACAGCACGCTTCATAA		
*sxtB*	SXTB-F	TTTGTAGGRCAGGCACTT	400	[53]
	SXTB-R	ATCATCGGTATCATCGGTAG		
*sxtM*	qMgrF	GAAGCACGAGTCAGCCTACA	129	[52]
	qMgrR	CAAAGCACCACCAGCCAAAA		
*sxtPer*	qPERgrF	CTGGGCGAGACATTTGAGA	116	[52]
	qPERgrR	GCACAGAGACAGGCGAACTA		
*sxtI*	OCT-F	TGCCGTTTTGTGCTTAGATG	923	[53]
	OCT-R	GGACGGAAGGACTCACGATA		
